# Value CMR: Towards a Comprehensive, Rapid, Cost-Effective Cardiovascular Magnetic Resonance Imaging

**DOI:** 10.1155/2021/8851958

**Published:** 2021-05-15

**Authors:** El-Sayed H. Ibrahim, Luba Frank, Dhiraj Baruah, V. Emre Arpinar, Andrew S. Nencka, Kevin M. Koch, L. Tugan Muftuler, Orhan Unal, Jadranka Stojanovska, Jason C. Rubenstein, Sherry-Ann Brown, John Charlson, Elizabeth M. Gore, Carmen Bergom

**Affiliations:** ^1^Medical College of Wisconsin, 8701 W Watertown Plank Rd, Milwaukee, WI 53226, USA; ^2^University of Wisconsin-Madison, 600 Highland Ave, Madison, WI 53792, USA; ^3^University of Michigan, 500 S State St., Ann Arbor, MI 48109, USA; ^4^Washington University, 1 Brookings Dr, St. Louis, MO 63130, USA

## Abstract

Cardiac magnetic resonance imaging (CMR) is considered the gold standard for measuring cardiac function. Further, in a single CMR exam, information about cardiac structure, tissue composition, and blood flow could be obtained. Nevertheless, CMR is underutilized due to long scanning times, the need for multiple breath-holds, use of a contrast agent, and relatively high cost. In this work, we propose a rapid, comprehensive, contrast-free CMR exam that does not require repeated breath-holds, based on recent developments in imaging sequences. Time-consuming conventional sequences have been replaced by advanced sequences in the proposed CMR exam. Specifically, conventional 2D cine and phase-contrast (PC) sequences have been replaced by optimized 3D-cine and 4D-flow sequences, respectively. Furthermore, conventional myocardial tagging has been replaced by fast strain-encoding (SENC) imaging. Finally, T1 and T2 mapping sequences are included in the proposed exam, which allows for myocardial tissue characterization. The proposed rapid exam has been tested in vivo. The proposed exam reduced the scan time from >1 hour with conventional sequences to <20 minutes. Corresponding cardiovascular measurements from the proposed rapid CMR exam showed good agreement with those from conventional sequences and showed that they can differentiate between healthy volunteers and patients. Compared to 2D cine imaging that requires 12-16 separate breath-holds, the implemented 3D-cine sequence allows for whole heart coverage in 1-2 breath-holds. The 4D-flow sequence allows for whole-chest coverage in less than 10 minutes. Finally, SENC imaging reduces scan time to only one slice per heartbeat. In conclusion, the proposed rapid, contrast-free, and comprehensive cardiovascular exam does not require repeated breath-holds or to be supervised by a cardiac imager. These improvements make it tolerable by patients and would help improve cost effectiveness of CMR and increase its adoption in clinical practice.

## 1. Introduction

Cardiac magnetic resonance imaging (CMR) is considered the gold standard for measuring heart function, including cardiac volumes and mass [1–4]. Furthermore, information about cardiac function, structure, tissue composition, and blood flow could be obtained in a single CMR exam. Compared to echocardiography, CMR has higher reproducibility, higher accuracy, and is not affected by the acoustic window, geometric assumptions, or operator's skills [5]. Nevertheless, CMR is underutilized due to long scanning times, the need for multiple breath-holds, the use of a contrast agent, and higher cost compared to echocardiography [6].

In this study, we propose a rapid CMR exam based on recent developments in imaging sequences. The proposed exam is both rapid and provides comprehensive cardiovascular information without the need for contrast agents or multiple breath-holds, which makes it cost-effective and allows for wider adoption in clinical practice. The proposed exam includes advanced sequences for evaluating global and regional cardiac functions and myocardial tissue characterization, as well as flow hemodynamics in the heart, valves, and large vessels. The information from different sequences complement each other to provide a complete picture about heart health. Time-consuming conventional sequences are replaced in the proposed exam by advanced sequences that are mostly free-breathing or real-time and do not require breath-holding, which resulted in total scan time of less than 20 minutes, versus more than 1 hour for conventional CMR.

Cine imaging is an essential part of almost every CMR exam, which is used to evaluate the heart function based on basic cardiovascular measurements such as volumes, mass, and ejection fraction (EF) [3]. Nevertheless, conventional cine imaging requires long scan times due to the need to acquire a stack of short-axis (SA) slices (typically 12-16 slices) that covers the whole heart, in addition to other long-axis (LA) slices. As cine images are typically acquired with one slice per breath-hold, acquiring all cine images in this conventional fashion consumes a considerable amount of scan time. In the proposed rapid exam, we replaced conventional breath-holding 2D cine imaging with a single 3D-cine scan [7, 8] based on recent developments in acquisition and reconstruction techniques [9–12], which require only 1-2 breath-holds and result in 80-90% saving in scan time compared to 2D acquisition. We further evaluated the effects of different imaging acquisition strategies on image quality and resulting clinical measurements in volunteer scans.

CMR blood flow imaging is essential in certain applications, e.g., in valvular and congenital heart diseases. It also provides important information about hemodynamic patterns, diastolic function, and vessel wall stiffness in different cardiovascular diseases. Typically, 2D phase-contrast (PC) sequences are used for flow imaging, where each slice is prescribed and acquired in a separate breath-hold. This leads to long scan times with the possibility of scan repetition to adjust slice positioning, since accurate plane prescription is necessary for quantitative flow analysis. In the proposed exam, 2D PC flow imaging was replaced by an advanced 4D-flow imaging [13, 14] based on recent developments in acquisition and reconstruction techniques [9, 12], which allows for free-breathing acquisition, whole-chest coverage, and inline image reconstruction in ~10 minutes, independent of the patient's breathing pattern. This approach significantly reduces scan time and allows for measuring flow and other derivative parameters at any location in the acquired volume without the need to repeat scans. In this study, we compared flow measurements from 4D-flow acquisition to those from conventional 2D PC flow imaging at different locations and show the capability of 4D flow for differentiating hemodynamics between healthy volunteers and patients.

Measuring regional cardiac function parameters, e.g., myocardial strain, has been shown to be valuable for early detection of subclinical cardiac dysfunction before the global function, e.g., EF is affected [15, 16]. CMR tagging is typically used for strain imaging [17, 18]. Nevertheless, the tagging sequence requires a separate breath-hold for each acquired slice, which makes it time consuming. Furthermore, the resulting tagged images have limited spatial resolution, depending on tag separation, and require specialized analysis software. In the proposed exam, CMR tagging was replaced by fast strain-encoding (SENC) imaging [19, 20], which has been previously validated against conventional tagging in several studies [21]. Compared to CMR tagging, SENC imaging has the following advantages: (1) real-time (one slice per heartbeat) acquisition, (2) high spatial resolution, and (3) instant, simple postprocessing that results in color-coded strain maps, replacing tagging with SENC results in significant savings in scan time.

Finally, the proposed exam includes T1 and T2 mapping [22, 23] sequences for myocardial tissue characterization. The advantages of these techniques are that they provide quantitative measurements of myocardial T1 and T2 relaxation parameters without the need for a contrast agent. Elevated T1 and T2 values have been previously shown to be associated with the presence of tissue fibrosis and edema, respectively [22, 24].

## 2. Materials and Methods

Human studies were conducted with the approval of the Institutional Review Board. Two healthy subjects were scanned on a 3T MRI scanner (GE Healthcare, Waukesha, WI) equipped phased-array coil. The proposed rapid CMR exam included the following sequences: 3D-cine (full heart coverage in 1-2 breath-holds), navigator-echo-free 4D-flow (whole-chest coverage in ~10 minutes of free-breathing), SENC (3 SA and 3 LA slices obtained in 6 heartbeats without breath holding), and T1 (Modified Look-Locker Inversion recovery (MOLLI)) [25] and T2 (multiecho fast spin echo (MEFSE)) mappings, with the same coverage as in SENC imaging. The two volunteers were also imaged using conventional CMR sequences to compare results to those from the rapid exam. Furthermore, four patients newly diagnosed with lung cancer or sarcoma were imaged using advanced techniques proposed in this protocol to evaluate hemodynamics and myocardial tissue characterization and compare the results to those from volunteers.

The implemented 3D cine imaging technique included a number of acquisition and reconstruction developments, including variable-density *k*-*t* sampling [12] and kat-ARC acceleration (autocalibrating reconstruction for Cartesian sampling with *k*- and adaptive-*t*-space data synthesis; a spatiotemporal correlation-based autocalibrating parallel imaging method) [9–11]. The technique allows for an acceleration factor of 9 and whole-heart imaging in 1-2 breath-holds. The following acquisition strategies were implemented with whole-heart coverage: (1) CONV: conventional 2D cine acquisition with SA slices acquired in separate breath-holds; (2) 2-SA: two overlapping 3D-cine slabs acquired in the SA direction in two breath-holds; (3) 1-SA: one 3D-cine thick slab acquired in the SA direction in a single breath-hold; (4) 2-AXIAL: two overlapping 3D-cine slabs acquired in the axial direction (with almost isotropic resolution) in two breath-holds; and (5) 1-AXIAL: one 3D-cine thick slab acquired in the axial direction (with almost isotropic resolution) in a single breath-hold. The imaging parameters for 2D cine imaging were repetition time (TR) = 3.6 ms, echo time (TE) = 1.3 ms, flip angle = 55°, and views per segment = 14. The imaging parameters for 3D-cine sequence were TR = 3 ms, TE = 1.2 ms, flip angle = 40°, and views per segment = 20. Both 2D cine and 3D-cine sequences were obtained with fast imaging employing steady-state acquisition (FIESTA) acquisition, number of averages = 1, and readout bandwidth = 488 Hz/pixel. Additional information about spatial and temporal resolutions of different acquisitions is shown in Table 1.

The cine images were processed using the cvi^42^ software (Circle Cardiovascular Imaging, Calgary, Canada). The AXIAL datasets were reformatted to generate a stack of SA and LA slices. The SA and LA images from different acquisition scenarios were semiautomatically processed to measure left ventricular (LV) volumes, EF, and mass, and EF. The myocardium signal-to-noise ratio (SNR) was measured as the ratio between mean myocardial signal intensity and background standard deviation (SD), as previously illustrated [26]; although, it should be noted that this approach could lead to measurements overestimation [27]. The blood-to-myocardium contrast-to-noise ratio (CNR) was measured as the ratio between the mean blood-to-myocardial signal intensity difference and background SD. For myocardium signal intensity measurement, elliptical regions of interest (ROIs), 0.5-1 cm^2^ each, were selected in the septal wall of the acquired (or reformatted) slices in the short-axis stack of each cine acquisition at end systole. For blood signal intensity measurement, elliptical ROIs, 1-4 cm^2^ each, were selected in the LV blood pool in the same images used for myocardium signal intensity measuring, such that the ROIs cover large area of blood without including the papillary muscles. For background signal intensity measurement, elliptical ROIs, 3-4 cm^2^ each, were selected in anterior nontissue (air) regions in the same images used for myocardium signal intensity measuring, such that the ROIs do not include artifacts.

The implemented 4D-flow imaging technique utilized several acquisition and reconstruction developments, including variable-density *k*-*t* sampling (increasing signal averaging at the k-space center) for acquisition-efficient motion suppression [12], kat-ARC acceleration [9], and overlapping multislab acquisition for inflow blood signal enhancement without using a contrast agent. The technique allows for an acceleration factor of 8, which enabled navigator-echo-free, whole-chest coverage in about 10 minutes. The 4D-flow imaging parameters were as follows: 3D time-resolved gradient echo sequence, 3 slabs with 32 slices per slab, and 5 overlapping slices between adjacent slabs, matrix = 180 × 180, field of view (FOV) = 360 × 360 mm^2^, spatial resolution = 2 × 2 × 2.4 mm^3^, views per segment = 4, number of heart phases = 20, flip angle = 8°, readout bandwidth = 488 Hz/pixel, acceleration factor = 8, velocity encoding (venc) = 160 cm/s, and number of reconstructed images ~12,500. Conventional 2D PC flow images were acquired at seven locations (for comparison with 4D-flow): ascending aorta, proximal and distal descending aorta, main and right pulmonary arteries, and mitral and tricuspid valves. The 2D PC flow imaging parameters were as follows: gradient echo sequence, TR = 6 ms, TE = 3.8 ms, matrix = 192 × 160, FOV = 360 × 360 mm^2^, slice thickness = 6 mm, readout bandwidth = 488 Hz/pixel, flip angle = 25°, views per segment = 6, venc = 160 cm/s, numbe of heart phases = 30, and breath − holding time (not including recovery time inbetween repeated breath − holds) = 15 s per slice.

The 2D PC and 4D-flow images were processed using the cvi^42^ software, where the 4D-flow images were analyzed to measure blood flow in the same planes acquired with the 2D PC flow imaging sequence. The 4D-flow images were analyzed on a DELL Precision 7540 mobile workstation (laptop) equipped with 8-core Xeon processor, 128 GB RAM, and NVIDIA Quadro RTX 3000 card, which resulted in streamlined analysis without noticeable time lags. The following parameters were measured at different locations: blood flow, velocity, volume, vascular pressure gradient, valvular regurgitation fraction, forward flow time, and ventricular early-to-atrial filling ratio (E/A) in the left and right ventricles.

SENC imaging is based on modulating the magnetization in the through-plane (slice selection) direction and then acquiring two sets of time-resolved images with different demodulation frequencies, from which tissue strain in the through-plane direction can be measured [19]. The implemented SENC sequence included a number of techniques that allowed for reducing scan time to one slice per heartbeat, including localized excitation, fast readout, and interleaved image acquisition [20]. Localized excitation is implemented by replacing one of the SENC modulation hard radiofrequency (RF) pulses by a slice-selective pulse in the phase-encoding direction in order to reduce the excited FOV and thus use a smaller acquisition FOV (and matrix) to accelerate acquisition without introducing aliasing artifacts. The two sets of demodulated images (called low-tuning (LT) and high-tuning (HT) images, which acquire data at low-frequency and high-frequency, respectively) are acquired in an interleaved fashion in a single heartbeat. The SENC images were analyzed to measure circumferential (Ecc) and longitudinal (Ell) strains. Conventional tagging images were acquired for reference with the following imaging parameters: TR = 5.7 ms, TE = 3.1 ms, flip angle = 8°, slice thickness = 7 mm, matrix = 212 × 192, FOV = 360 × 360 mm^2^, number of averages = 1, readout bandwidth = 391 Hz/pixel, tag spacing = 7 mm, number of heart phases = 30, and scan time = 12 s per slice.

The imaging parameters of the MOLLI T1 mapping sequence were as follows: 8 images acquired using the 5(3 s)3 acquisition pattern, FIESTA sequence, TR = 2.9 ms, TE = 1.3 ms, flip angle = 35°, slice thickness = 8 mm, matrix = 160 × 148, FOV = 360 × 360 mm^2^, number of averages = 1, readout bandwidth = 977 Hz/pixel, and scan time = 11 s per slice. The imaging parameters of the MEFSE T2 mapping sequence were as follows: TR = 895 ms, TE = 11 − 77 ms (4 echoes with 22 ms increments), echo train length (ETL) = 16, flip angle = 90°, slice thickness = 8 mm, matrix = 180 × 180, FOV = 360 × 360 mm^2^, number averages = 1, readout bandwidth = 651 Hz/pixel, and scan time = 16 s per slice. Both MOLLI and MEFSE images were analyzed using the cvi^42^ software to generate T1 and T2 maps, respectively. Bland-Altman [28] analysis was conducted to assess intermethod variability between different acquisition techniques. Student's *t*-test was used to measure significance of measurement differences between volunteers and patients (*P* < 0.05 considered significant).

## 3. Results and Discussion

### 3.1. Results

**Figure 1:**
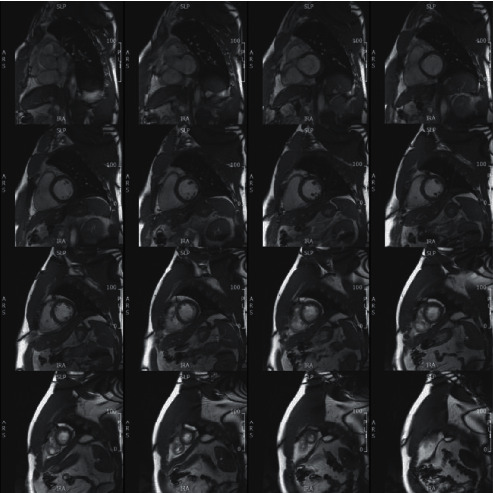
Conventional cine acquisition. A stack of short-axis slices covering the heart from base (top left) to apex (bottom right), obtained using conventional 2D cine acquisition, where each slice is acquired in a separate breath-hold. Total scan time (including recovery time in − between consecutive breath − holds) = 270 s.

**Figure 2:**
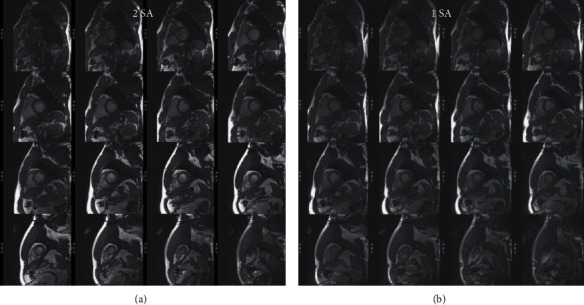
3D cine acquisition in the short-axis direction. Stacks of short-axis slices covering the heart from base (top left) to apex (bottom right), obtained in the same subject as in Figure 1 using accelerated 3D-cine acquisition with 2 overlapping short-axis (SA) slabs (a) and one thicker SA slab (b). Scan time is 48 s and 20 s for the 2-slabs and 1-slab acquisitions, respectively.

**Figure 3:**
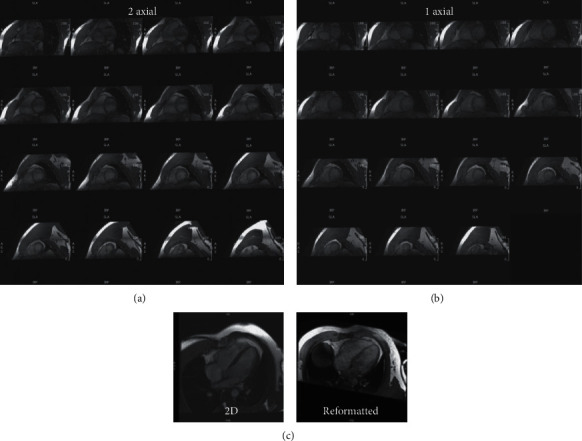
3D cine acquisition in the axial direction. Reformatted short-axis slices, covering the heart from base (top left) to apex (bottom right), obtained in the same subject as in Figures 1 and 2 using accelerated semi-isotropic 3D-cine acquisition with 2 overlapping axial slabs (a) and one thicker axial slab (b). Scan time is 48 s and 21 s for the 2-slabs and 1-slab axial acquisitions, respectively. (c) Four-chamber slice reformatted from the semi-isotropic 3D-cine acquisition (right) and a corresponding slice acquired using conventional 2D cine imaging (left).

Scanning time was 18-20 minutes for the rapid exam and 1-1¼ hours for the conventional exam. Cine images from the same subject were acquired using different techniques that are shown in Figures 1–3.  Table 1 summarizes scan time, SNR, and CNR results of different cine acquisitions. The 2-SA, 1-SA, 2-AXIAL, and 1-AXIAL 3D cine acquisitions resulted in 82%, 93%, 82%, and 92% savings in scan time, respectively, compared to conventional 2D cine acquisition. SNR slightly changed between different acquisitions, while CNR decreased by 28%, 46%, 78%, and 81% in the 2-SA, 1-SA, 2-AXIAL, and 1-AXIAL 3D cine acquisitions, respectively. Most of the measurements' differences between 2D acquisition and 3D acquisitions lied within the 2 standard deviation agreement limit in the Bland-Altman analysis.

**Figure 4:**
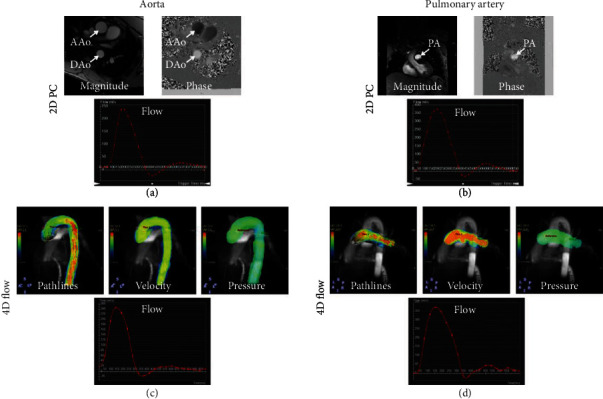
2D phase-contrast and 4D-flow imaging in large arteries. Representative 2D phase contrast (PC) images and flow curves (a, b) and the corresponding 4D-flow maps and curves (c, d) at different measurement sites of the aorta (a, c) and pulmonary artery (b, d). The images show good agreement between flow curves by the two techniques, in addition to the extra information and (pathlines, velocity, and pressure maps) provided by 4D-flow acquisition. AAo: ascending aorta; DAo: descending aorta; PA: pulmonary artery.

**Figure 5:**
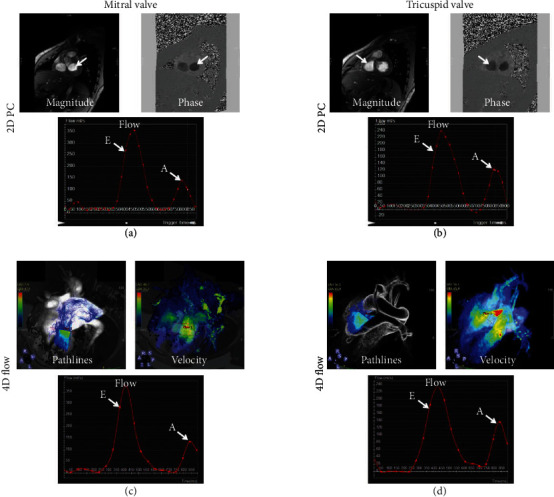
2D phase-contrast and 4D-flow valvular imaging. Representative 2D phase contrast (PC) images and flow curves (a, b) and the corresponding 4D-flow maps and curves (c, d) at measurement sites across the mitral valve (a, c) and the tricuspid valve (b, d). The images show good agreement between flow curves by the two techniques, in addition to the extra information (pathlines and velocity maps) provided by 4D-flow acquisition. E: early filling peak; A: atrial filling peak.

**Figure 6:**
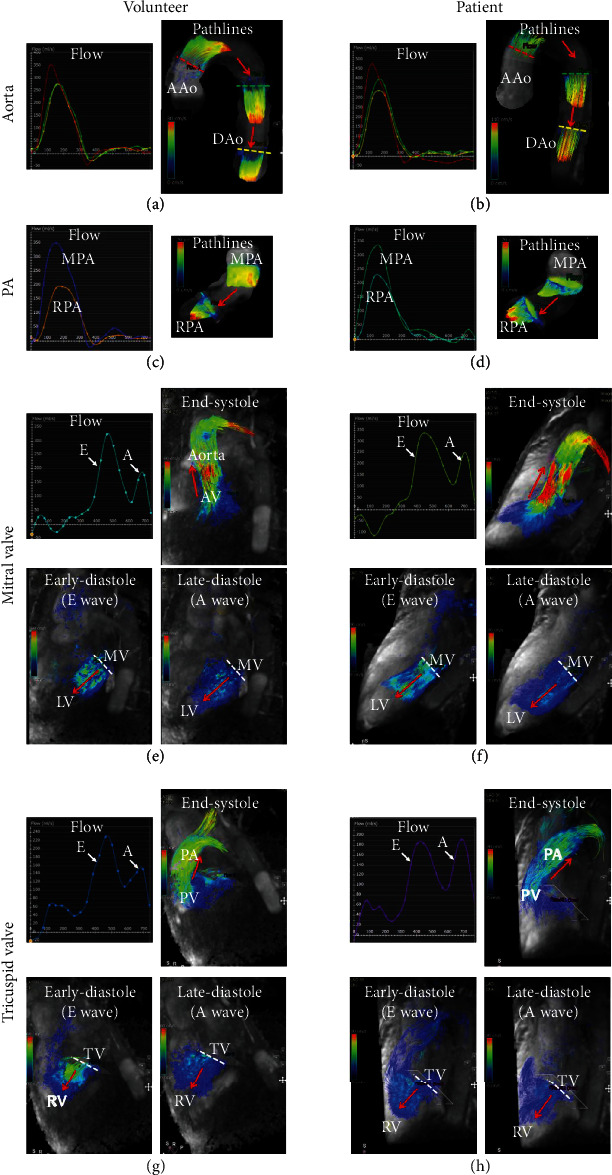
Comparison between volunteer (a, c, e, g) and patient (b, d, f, h) flow dynamics in the aorta (a, b), pulmonary artery (c, d), mitral valve (e, f), and tricuspid valve (g, h). The figure shows different patterns of flow curves and hemodynamics between the volunteer and patient; for example, the early-to-atrial (E/A) filling ratios in the mitral and tricuspid valves are lower in patient compared to volunteer, which may reflect diastolic dysfunction. Flow directions are shown by red arrows. 4D-flow videos in patient and volunteer are provided in supplementary materials (available here). AAo: ascending aorta; DAo: descending aorta; PA: pulmonary artery; MPA: main pulmonary artery; RPA: right pulmonary artery; LV: left ventricle; RV: right ventricle; AV: aortic valve; PV: pulmonary valve; MV: mistral valve; TV: tricuspid valve.

Figures 4 and 5 show 2D PC flow results and the corresponding 4D-flow maps and flow curves at different measurement sites of large arteries (aorta and pulmonary artery) and atrioventricular valves (mitral and tricuspid), respectively. All scans were successfully completed without motion or other imaging artifacts. Hemodynamic measurements by 2D PC flow and 4D-flow techniques were acquired at seven sites: ascending, descending, and distal aorta; main and right pulmonary arteries; and mitral and tricuspid valve. All measurements' differences between 2D PC and 4D-flow acquisitions lied within the 2 standard-deviation agreement limit in the Bland-Altman analysis.  Table 2 and Figure 6 show comparison of hemodynamic results from volunteers and patients. The measurements showed differences between the two groups, where aortic flow, pulmonary artery flow, and E/A ratio showed statistically significant differences.

**Figure 7:**
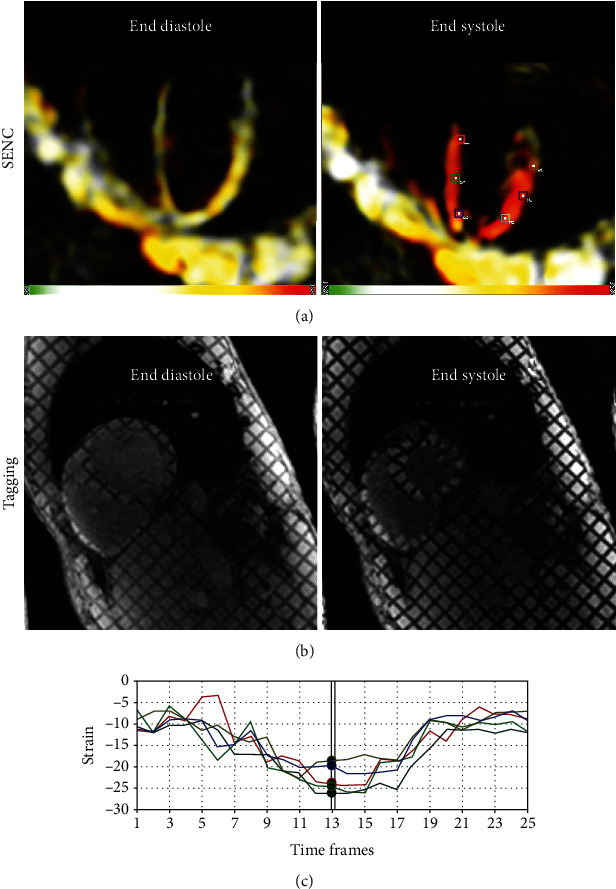
Strain-encoding imaging. Representative strain-encoding (SENC) (a) and reference tagging (b) images acquired at both end diastole and end systole in the same subject. Note: increased strain resolution (at the pixel level) and intuitive color-coded strain maps in SENC imaging compared to conventional tagging. (c) Circumferential strain curves generated from the SENC images at different positions shown by the markers in (a).

**Figure 8:**
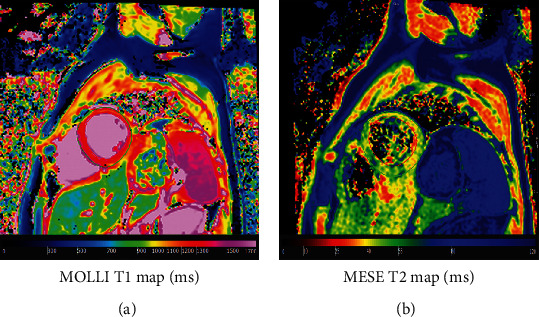
T1 and T2 mappings. Modified Look-Locker Inversion recovery (MOLLI) T1 map (a) and multiecho fast spin echo (MEFSE) T2 map (b) acquired in a healthy volunteer. Myocardial T1 and T2 values lie within normal range as shown by the color bars.

Figure 7 shows representative SENC images and generated strain curves. The results showed normal strain values in the imaged volunteers: circumferential strain = −23.5 ± 2.1% and longitudinal strain = −19.7 ± 1.9%. Corresponding tagged images are shown in the figure for reference.  Figure 8 shows resulting T1 and T2 maps, where the T1 and T2 values were within normal range in the imaged volunteers: average T1 = 1233 ± 221 ms and T2 = 48.5 ± 1.7 ms.

## 4. Discussion

The proposed rapid CMR exam took less than 20 minutes, did not require contrast, and resulted in comprehensive and accurate measurements of global and regional cardiac functions, hemodynamic parameters in the heart and large arteries, and T1 and T2 maps. The proposed exam makes the scan protocol more easily tolerated by patients, cost-effective, and valuable from a clinical perspective.

The goal of this study was to evaluate the feasibility of the proposed rapid, comprehensive CMR exam based on advanced imaging techniques. Although small number of subjects were included in this study, the preliminary results demonstrate the potential of advanced acquisition strategies to reduce the scan times for a complete CMR protocol by about 70%. Despite the necessary tradeoffs in spatial/temporal resolutions and CNR with the proposed protocol, sufficient image quality was maintained such that the resulting clinical measurements were not significantly different from those obtained using conventional 2D techniques.

The single breath-hold 3D-cine acquisition, which is more feasible for smaller subjects, further reduces total scan time and avoids slice misregistration errors. Nevertheless, in patients with limited breath-holding capability, the single breath-hold can be split in 2 shorter breath-holds for 2-slab acquisition. AXIAL 3D acquisition saves more scan time, as no additional LA slices need to be acquired (they are rather reformatted from the acquired semiisotropic 3D dataset), and no experience is needed for slice prescription (the operator only needs to place an axial box covering the heart). Nevertheless, it should be noted that AXIAL 3D acquisition has reduced blood-to-myocardium CNR compared to SA 3D acquisition due to the time-of-flight effect in the latter when fresh unsaturated blood spins flow perpendicular to and into the SA slab, resulting in brighter blood signal and therefore higher blood-to-myocardium CNR. Furthermore, switching from two (overlapping) thinner slab acquisition to one thicker slab acquisition causes more reduction in CNR due to the reduced time-of-flight effect as blood travels a larger distance through the thicker slab and thus becomes more saturated and has lower signal. It should be noted that compressed-sensing-based single-heartbeat cine imaging has been recently introduced [29], which could have been used in place of conventional 2D segmented cine acquisition with significant saving in cine scan time as well.

The implemented accelerated 4D-flow technique allows for acquiring detailed whole-chest hemodynamic information in about 10 minutes. Measurements from accelerated 4D-flow were in good agreement with conventional 2D PC flow imaging. As motion correction is not based on navigator-echo tracking [12], the imaging time is known in advance and is not dependent on the patient's breathing pattern, which allows for efficient use of the scan time and the capability of adjusting the imaging parameters to reduce the 4D-flow scan time to a specified time duration. 4D-flow imaging has several advantages compared to 2D PC flow imaging. Besides reduction in total scan time and alleviating the need for repeated breath-holds and possible scan repetitions, prescription of the 4D-flow scan is quite simple (only placing a box around the heart), and therefore, compared to 2D PC flow imaging, no cardiac-specialist scanner operator is needed to run the 4D-flow sequence. Further, postprocessing and image analysis can be done at any plane of interest and are not restricted to the slices acquired during the CMR exam as in 2D PC flow imaging. Besides its short acquisition time, the short reconstruction time (~12,500 images are reconstructed inline within a couple of minutes) makes the scan feasible for clinical adoption as other sequences can be run on the scanner after the 4D-flow data acquisition without congesting the reconstruction pipeline. Finally, with the computational power available today and cloud computing options, analysis of 4D-flow dataset can be done in real time without experiencing time lags, as illustrated in this study.

One key advantage of the proposed rapid exam is using SENC for fast strain measurements in only few seconds. A large body of literature showed the value of myocardial strain analysis for early detection of subclinical cardiac dysfunction in different cardiovascular diseases before the global heart function is affected or symptoms of cardiovascular diseases are observed [30–32], especially in coronary artery disease [33] and heart failure [34], which would allow for early intervention and better outcomes. Further, besides providing strain measurements, SENC showed the capability of producing reproducible global function measurements that are in close agreement with those obtained from cine images [35–37]. Therefore, for further reduction of scan time, more SENC images (a total of 6 SA and 6 LA slices) could be acquired to provide volumes and mass measurements in lieu of using cine imaging.

The optimized proposed fast CMR exam showed the capability of differentiating between measurements from volunteers and patients. Although only few measurements reached statistical significance level for the differences between the two groups, the measurements provide clinical insights about the differences between the two groups. For example, the differences in flow and velocity measurements between the two groups at different sites reflect undergoing changes in hemodynamic patterns. This was noticeably shown by the reduced E/A ratios measured through the mitral and tricuspid valves in the patients compared to volunteers, which reflect undergoing diastolic dysfunction in the patients. Furthermore, myocardial T1 and T2 values were slightly elevated in the patients, which may imply undergoing changes in myocardial tissue composition, e.g., increased diffused fibrosis and edema. It should be noted that although the imaged patients were newly diagnosed with cancer without major cardiovascular diseases, it is not uncommon for cancer patients to have cardiovascular risk factors, which may explain the differences in patients' measurements compared to those from healthy volunteers.

It should be noted that other fast and valuable CMR sequences have been recently developed, which could be added to the proposed rapid CMR exam to provide more information with only slight increases in scan time. One such example is myocardial blood oxygenation level dependent- (BOLD-) based imaging [38, 39], which can provide useful information about perfusion and myocardial vasodilation after hyperventilation maneuvers and without the need for exogeneous contrast agent or pharmaceutical stress agents [38]. Therefore, adding the BOLD sequence to the proposed exam would add one more dimension, in addition to T1 and T2 mappings, for tissue characterization.

Development of this shorter and more cost-effective method for CMR may be particularly helpful for cancer patients and survivors, especially those at high risk of developing cardiovascular complications from cancer therapy. For example, a myriad of pharmacologic treatments in cancer can lead to cardiomyopathy, which is often heralded by changes in myocardial strain. Offering a short, cost-effective, and accurate CMR option will add to the toolbox for cardiologists who care for these patients in order to screen for early evidence of subclinical toxicity, in order to preempt and prevent overt cardiomyopathy and clinical heart failure. Thus, application of these new CMR methods may enhance clinical care.

One limitation of the proposed rapid exam is that it does not include perfusion or late gadolinium enhancement (LGE) sequences for ischemia and viability imaging, respectively. Nevertheless, both perfusion and LGE sequences require contrast administration and increase the scan time, especially with the delay time post contrast injection and before LGE imaging. Furthermore, with recent concerns about the side effects of gadolinium-based contrast agents and their accumulation in different tissues, efforts are being directed towards wider adoption of contrast-free CMR sequences in clinical practice [40]. It should be noted that some noncontrast-enhanced sequences showed the capability of identifying different tissue types, which could help with tissue characterization. For example, strain imaging has the capability to differentiate between viable and infarcted myocardium [41–43] and to identify ischemic defects [44, 45] with results in agreement with LGE and perfusion images, respectively. Furthermore, fibrosis and myocardial infarction have higher T1 values compared to normal myocardium [22, 24]; therefore, the combination of functional, T1, and T2 mappings [23] could allow for tissue characterization without the need for gadolinium. Another limitation of this work is inability to conduct thorough statistical analysis due to the limited number of scanned subjects. However, the results showed the capability of advanced CMR techniques for differentiating between measurements from volunteers and patients. Therefore, the goal of this study was to show the feasibility of the proposed rapid CMR exam and its advantages compared to conventional CMR sequences, which warrants larger studies to confirm the value of the proposed rapid CMR exam and illustrate its clinical usefulness in different patient populations.

## 5. Conclusions

The proposed CMR exam is rapid and contrast-free and provides comprehensive information about the cardiovascular system, including early development of subclinical cardiac dysfunction. Furthermore, as advanced free-breathing, real-time, and 3D sequences are used in the proposed rapid exam, no repeated breath-holds nor specific plane prescription is needed, which increases patient comfort and allows noncardiac-experienced scanner operators to run the exam. These features are essential for improving cost-effectiveness of CMR and increasing its adoption in clinical practice, especially in asymptomatic patients with cardiovascular risk factors or patients at risk of developing heart failure who cannot withstand a long CMR exam.

## Figures and Tables

**Table 1:** Characteristics of different cine acquisition strategies.

Method	#stacks × #slices (× overlaps)	Spatial resolution (mm)	Temporal resolution (ms)	Myocardium SNR (mean ± SD)	Blood-to-myocardium CNR (mean ± SD)	Total scan time (s)	% time saving
CONV	16 2D slices	1.8 × 1.8 × 8	27	29 ± 7	69 ± 14	270	—
2-SA	2 × 14 × 4	1.8 × 1.8 × 7	40	32 ± 8	50 ± 21	48	82%
1-SA	1 × 20	1.8 × 1.8 × 7	40	29 ± 9	37 ± 15	20	93%
2-AXIAL	2 × 34 × 8	2.5 × 2.5 × 2.8	40	36 ± 11	15 ± 7	48	82%
1-AXIAL	1 × 46	2.5 × 2.5 × 2.8	40	33 ± 8	12 ± 3	21	92%

Abbreviations: CONV: conventional 2D cine acquisition; 2-SA: 3D-cine acquisition of two overlapping slabs in the short-axis (SA) direction; 1-SA: 3D-cine acquisition of one thick slab in the SA direction; 2-AXIAL: 3D-cine acquisition of two overlapping slabs in the axial direction with semi-isotropic resolution; 1-AXIAL: 3D-cine acquisition of one thick slab in the axial direction. Shown scan times include recovery time inbetween repeated breath-holds. CNR: contrast-to-noise ratio; SNR: signal-to-noise ratio.

**Table 2:** Measurements (mean ± SD) of different cardiovascular parameters in volunteers and patients. Asterisks indicate statistical significance (*P* < 0.05).

Parameter	Volunteers	Patients
AAo flow, max (ml/s)	423 ± 36	489 ± 71
AAo velocity, max (cm/s)	62 ± 17	51 ± 5
DAo flow, max (ml/s)	276 ± 4	373 ± 32
DAo velocity, max (cm/s)	65 ± 23	44 ± 10
Distal Ao flow, max (ml/s)^∗^	248 ± 1	340 ± 43
Distal Ao velocity, max (cm/s)	73 ± 16	37 ± 8
MPA flow, max (ml/s)	391 ± 20	415 ± 51
MPA velocity, max (cm/s)	55 ± 11	47 ± 4
RPA flow, max (ml/s)^∗^	167 ± 1	258 ± 26
RPA velocity, max (cm/s)	46 ± 5	29 ± 6
Mitral flow, max (ml/s)	340 ± 57	353 ± 42
Mitral velocity, max (cm/s)	26 ± 11	35 ± 7
Mitral early-to-atrial (E/A) filling ratio	2.2 ± 0.7	1.2 ± 0.4
Tricuspid flow, max (ml/s)	254 ± 28	286 ± 71
Tricuspid velocity, max (cm/s)	17 ± 6	25 ± 3
Tricuspid early-to-atrial (E/A) filling ratio^∗^	1.9 ± 0.3	0.8 ± 0.4
T1 (ms)	1233 ± 221	1287 ± 53
T2 (ms)	48.5 ± 1.7	53 ± 2.8

## Data Availability

The datasets used and/or analyzed during the current study are available from the corresponding author on reasonable request.

## Supplementary Materials

Supplementary Materials Video 1: 4D flow in a volunteer showing both systemic and pulmonary circulations in the heart and large vessels: https://drive.google.com/file/d/1fK4JbMoj5brCVoWm7mJdPsyDKuZ9eoaf/view?usp=sharing. Video 2: 4D flow in a patient showing both systemic and pulmonary circulations in the heart and large vessels: https://drive.google.com/file/d/1FM_-W9MPi_TnupUBgJWaocSHQRBlHKEc/view?usp=sharing.
