# Supplementary material for: Value CMR: Towards a Comprehensive, Rapid, Cost-Effective Cardiovascular Magnetic Resonance Imaging
Source: Int J Biomed Imaging. 2021 May 15;2021:8851958. doi: 10.1155/2021/8851958 (PMC8147553; doi:10.1155/2021/8851958)
Supplement: Supplementary Materials — Video 1: 4D flow in a volunteer showing both systemic and pulmonary circulations in the heart and large vessels: https://drive.google.com/file/d/1fK4JbMoj5brCVoWm7mJdPsyDKuZ9eoaf/view?usp=sharing. Video 2: 4D flow in a patient showing both systemic and pulmonary circulations in the heart and large vessels: https://drive.google.com/file/d/1FM_-W9MPi_TnupUBgJWaocSHQRBlHKEc/view?usp=sharing. [file 8851958.f1.zip › Supplementary materials.docx]

## Supplementary Materials

Video 1: 4D flow in a volunteer showing both systemic and pulmonary circulations in the heart and large vessels: <https://drive.google.com/file/d/1fK4JbMoj5brCVoWm7mJdPsyDKuZ9eoaf/view?usp=sharing>

Video 2: 4D flow in a patient showing both systemic and pulmonary circulations in the heart and large vessels. <https://drive.google.com/file/d/1FM_-W9MPi_TnupUBgJWaocSHQRBlHKEc/view?usp=sharing>

**NOTE: SUPPLEMENTARY VIDEO FILES ARE UPLOADED UNDER FIGURES AND TABLES SECTION.**
